# Targeted Heart Rate Control with Landiolol in Hemodynamically Unstable, Non-Surgical Intensive Care Unit Patients: A Comparative Study

**DOI:** 10.3390/medicina61091703

**Published:** 2025-09-19

**Authors:** Lyuboslav Katov, Jessica Gierak, Yannick Teumer, Federica Diofano, Carlo Bothner, Wolfgang Rottbauer, Karolina Weinmann-Emhardt

**Affiliations:** Ulm University Heart Center, Albert-Einstein-Allee 23, 89081 Ulm, Germany; lyuboslav.katov@uniklinik-ulm.de (L.K.); yannick.teumer@uniklinik-ulm.de (Y.T.); federica.diofano@uniklinik-ulm.de (F.D.); carlo.bothner@uniklinik-ulm.de (C.B.); wolfgang.rottbauer@uniklinik-ulm.de (W.R.)

**Keywords:** landiolol, critically ill patients, intensive care unit, ICU, atrial fibrillation, heart rate, blood pressure, standard therapy

## Abstract

*Background and Objectives:* Atrial fibrillation (AF) in critically ill patients (CIP) is associated with worse outcomes and increased mortality in the intensive care unit (ICU). Rhythm control strategies are often unfeasible due to underlying comorbidities, making rate control the preferred initial approach. However, conventional beta-blockers may worsen hemodynamics through negative inotropic effects and peripheral vasodilation. Landiolol, an ultra-short-acting adrenoreceptor antagonist, may offer an alternative due to its high β_1_-cardioselectivity and minimal blood pressure (BP) impact. This study evaluated the efficacy and feasibility of landiolol in hemodynamically unstable CIP with tachyarrhythmia, used as add-on therapy after failure of standard treatments. *Materials and Methods:* Ten CIP, admitted for non-postoperative reasons, were prospectively enrolled for landiolol treatment (L-group) in the ICU of Ulm University Heart Center between July and December 2017. The control group contained 41 patients who had received standard therapy without landiolol (NL-group). The primary composite endpoint was defined as heart rate (HR) reduction while maintaining mean arterial pressure (MAP) above 65 mmHg. *Results:* The most frequent reason for ICU admission was hemodynamic instability related to tachyarrhythmia in patients with cardiogenic or septic shock. At therapy initiation, all patients exhibited a compromised hemodynamic status, with a median MAP of 68.0 (IQR 60.0–80.0) mmHg and a median HR of 160.0 (IQR 144.0–176.0) bpm. After a three-hour observation period, no significant differences in BP values were observed between the groups. The primary composite endpoint was achieved at comparable rates in both groups (*p* = 0.525). However, patients in the L-group achieved a greater reduction in HR compared to those in the NL-group (25.3% vs. 21.9%, *p* < 0.001). *Conclusions:* Landiolol achieved more effective HR control than standard therapy without adversely affecting BP stability. These findings suggest that landiolol may be a feasible and effective option for HR control in ICU CIP.

## 1. Introduction

Atrial fibrillation (AF) has a high incidence in intensive care units (ICUs), with reported rates ranging from approximately 1.8% to 8.8% in non-cardiac ICUs [1,2,3]. Following cardiac surgery, its occurrence increases significantly, affecting up to approximately 65% of patients [4]. AF can cause significant complications in already high-risk intensive care patients due to its impact on hemodynamics, including rapid ventricular rates, reduced cardiac output, and an increased risk of thromboembolic events [5,6,7]. The mechanisms underlying both new-onset AF and the recurrence of prior AF during critical illness remain incompletely understood [6]. In the ICU setting, the factors contributing to the arrhythmogenic substrate and the specific triggers for AF are distinct from those observed in community-acquired AF. Acute pathophysiological events associated with critical illness, such as systemic infection and systemic inflammatory response, can lead to different shock states, ultimately accelerating processes like cardiac remodeling, atrial fibrosis and finally leading to the persistence of AF [8,9,10].

The primary therapy for AF is oral anticoagulation (OAC) to reduce the risk of thromboembolic events, particularly ischemic stroke. The duration of the OAC therapy depends on individual stroke risk stratification, commonly assessed using the CHA_2_DS_2_-VA score [11]. In hemodynamically unstable patients, electrical cardioversion is the first-line treatment to restore sinus rhythm (SR) [11,12]. For rhythm control, class Ic antiarrhythmic drugs (AAD), e.g., flecainide and propafenone, can be used for selected patients without structural heart disease [13,14] and class III antiarrhythmics, such as amiodarone, dronedarone and sotalol, are effective in maintaining SR but require careful monitoring due to potential proarrhythmic and extracardiac side effects [15]. Alternatively, rate control can be achieved using beta-blockers, non-dihydropyridine calcium channel blockers (e.g., verapamil, diltiazem), or cardiac glycosides [16,17].

Landiolol is an ultra-short-acting, β_1_-selective beta-blocker that undergoes rapid hydrolysis by esterases in the blood and liver, leading to a short elimination half-life (t_1_/_2_) of approximately 4 min in healthy individuals [18,19,20]. Clinical studies have demonstrated the feasibility and tolerability of landiolol therapy in patients with new-onset AF admitted to both non-surgical and surgical ICUs [21]. Furthermore, comparative data indicate that landiolol provides superior heart rate (HR) control compared with digoxin and esmolol in patients with AF and left ventricular dysfunction [22]. We investigated landiolol for acute HR control in critically ill, non-post-surgical medical patients with unstable hemodynamics after failure of standard therapy, using a matched index time point design and a three-hour hemodynamic follow-up. This approach provides new comparative insights into the efficacy, safety, and potential added benefit of landiolol over standard care in this patient group.

## 2. Materials and Methods

### 2.1. Study Design

Ten critically ill patients with tachycardia and hypotension were prospectively enrolled and treated with landiolol (L-group) in the ICU at Ulm University Heart Center between July and December 2017. A control group of 41 patients who received standard care without landiolol therapy (NL-group) was identified from the period September 2016 to December 2017 and specifically selected to match the L-group in terms of clinical characteristics and eligibility criteria. Both groups were recruited from a medical (non-surgical) intensive care unit, with no patients admitted due to surgical interventions. Identical inclusion and exclusion criteria were applied to both groups.

Eligibility for landiolol treatment included adult patients over 18 years who were admitted to the ICU and exhibited documented hemodynamic deterioration due to the acute onset of tachycardia. Inclusion required a diagnosis of either new-onset or pre-existing AF necessitating rate control due to a previously insufficient attempt to reduce HR or control rhythm using amiodarone, digoxin, electrical cardioversion, or the presence of contraindications to acute rhythm control (e.g., recent device implantation, intracardiac thrombus, etc.). Patients with normofrequent or hemodynamically non-relevant AF, known beta-blocker intolerance or allergy, as well as those with documented prior bradycardia or high-degree atrioventricular block, were excluded from the study. Informed consent was obtained before treatment initiation. This trial was approved by the local Ethics Committee of Ulm University and conducted in accordance with the principles outlined in the Declaration of Helsinki (German Clinical Trials Register ID: DRKS00013013, protocol 324/16, 12 October 2016). The delay in publication is mainly attributable to extended data analysis, regulatory approvals, and internal review processes.

### 2.2. Landiolol and Standard Treatment

In the L-group, continuous intravenous landiolol infusion was administered to achieve rapid HR control. The initial infusion rate was based on current recommendations and patient-specific hemodynamic conditions. Titration was performed according to the patient’s response, aiming for a target HR of <110 bpm while maintaining hemodynamic stability. The infusion was initiated at 1 μg/kg/min and adjusted as needed up to a maximum of 10 μg/kg/min. In both groups (L-group and NL-group), monitoring started from a clearly defined baseline time point: in the L-group, the baseline corresponded to the initiation of landiolol therapy after the failure of standard rate control measures. Failure of standard therapy was assessed clinically by at least one board-certified specialist in internal medicine and intensive care medicine. Based on this clinical judgment, the initiation of landiolol therapy was then individually decided. In the NL-group, an “index time point” was retrospectively determined by a board-certified specialist in internal medicine and intensive care medicine, identifying the moment after the initiation of standard therapy (e.g., amiodarone, digoxin) when the treatment was judged to have failed due to an insufficient HR response and where landiolol would have been considered if available. From this baseline time point (either landiolol start or index time), HR, systolic blood pressure, diastolic blood pressure, and mean arterial pressure (MAP) were monitored and recorded at 0, 30, 60, 90, 120, and 180 min. Data were collected continuously and documented at these specific intervals to allow direct comparison between groups.

### 2.3. Primary Composite Endpoint

The primary endpoint of this study was the achievement of adequate HR control within two hours after landiolol administration or index time point, defined as a reduction of at least 20% from baseline HR or an absolute HR < 100 bpm while maintaining stable hemodynamics (MAP > 65 mmHg without the need for additional vasopressors).

### 2.4. Secondary Endpoint

Secondary endpoints included changes in systolic, diastolic, and MAPs within three hours of landiolol administration or after index time point, as well as the incidence of bradycardia (<50 bpm) or hypotension (MAP < 65 mmHg) requiring discontinuation of landiolol.

### 2.5. Statistical Analysis

All statistical analyses were performed using SPSS Statistics (Version 29, IBM, Armonk, NY, USA). Continuous variables were analyzed with the Mann–Whitney U test and expressed as median with interquartile range (IQR) or as mean ± standard deviation (SD). Categorical variables were examined with Fisher’s exact test and reported as absolute numbers and percentages. Multivariate logistic regression analysis was performed to identify potential predictors for achieving the primary composite endpoint. Statistical significance was defined as a *p*-value < 0.05.

## 3. Results

### 3.1. Baseline Characteristics

A total of 51 patients were included in the analysis, of whom 10 received therapy with landiolol (L-group), while the remaining 41 were treated with conventional therapy without landiolol (NL-group). Of all patients, 22 (43.1%) were female. The median age was slightly higher in the NL-group, though this difference was not statistically significant (*p* = 0.392). A normal left ventricular ejection fraction (LVEF) was observed in 23 patients (45.1%). There were no significant differences between the groups in patients with mildly (*p* = 1.000), moderately (*p* = 0.250), and severely reduced LVEF (*p* = 0.705). The most common reasons for intensive care unit (ICU) admission were septic shock (68.6%) and cardiogenic shock (33.3%), with no significant differences in APACHE II score at the admission between the groups (*p* = 0.930). Paroxysmal AF was more frequent in the L-group (*p* = 0.050), with no differences between the other AF types. Detailed baseline characteristics are summarized in Table 1.

### 3.2. Vital Parameters at ICU Admission

At ICU admission, the median (IQR) HR was 160.0 (142.0–175.0) beats per minute, and the median (IQR) respiratory rate was 24.0 (20.0–33.3) breaths per minute. Patients had a median (IQR) PaO_2_ of 86.0 (74.0–100.0) mmHg and a median (IQR) Horovitz index of 155.0 (113.7–215.0). There were no statistically significant differences between the groups in terms of vital parameters at ICU admission (Table 2).

### 3.3. Conventional Therapy

Approximately 70% of the patients received catecholamine therapy, mainly norepinephrine (62.7%) and to a lesser extent epinephrine (7.8%). 20 patients (39.2%) underwent unsuccessful electrical cardioversion. In the NL-group, 21 patients (51.2%) were treated with intensified infusion therapy, whereas this was the case in 1 in 10 patients (10.0%) in the L-group (*p* = 0.030). Amiodarone was administered to half of the patients, with no significant differences observed between the groups. The remaining conventional medications administered are presented in Table 3.

Out of the whole cohort, 23 patients (46.9%) underwent more than one treatment. Triple therapy with amiodarone, digoxin, and electrical cardioversion was attempted in 5 patients (12.2%). In the L-group, 6 patients (60%) were treated with a combination of electrical cardioversion and amiodarone. Despite these measures, adequate rate control was not achieved in 20 patients (48.8%) in the NL group.

### 3.4. Hemodynamic Changes Within the First Three Hours of Treatment

**Figure 1:**
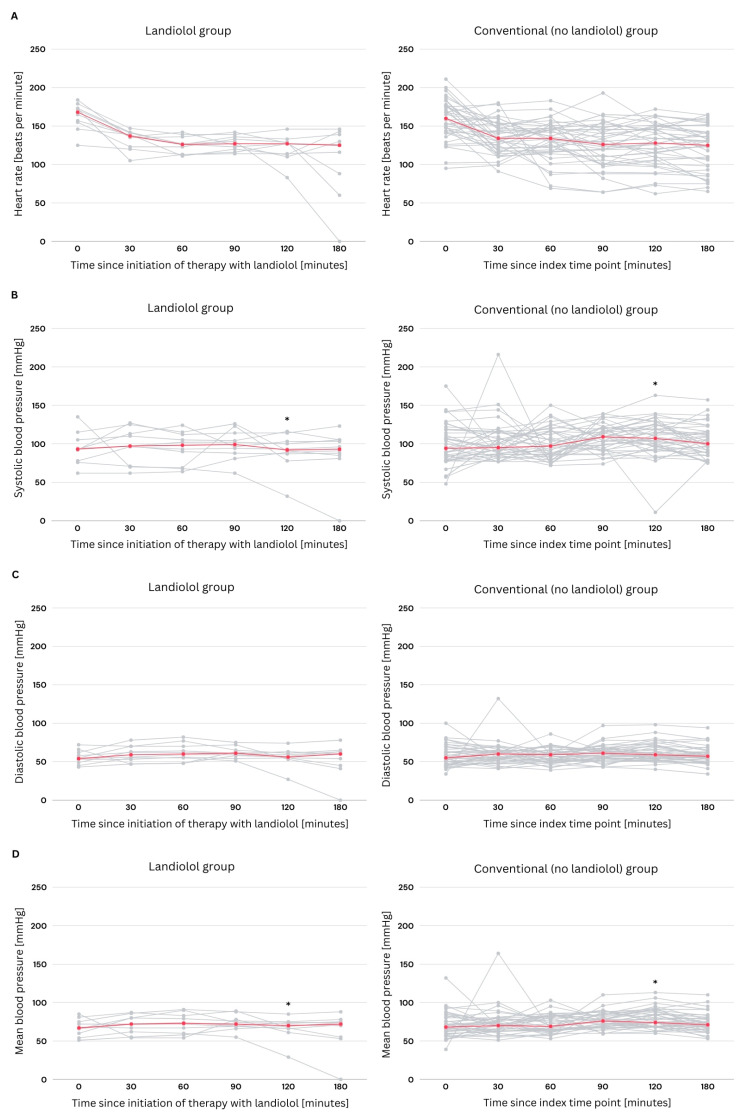
Line charts illustrating heart rate and blood pressure measurements over time in the landiolol group (L-group) and the standard therapy group (NL-group). (**A**) Heart rate (beats per minute), (**B**) systolic blood pressure (mmHg), (**C**) diastolic blood pressure (mmHg), and (**D**) mean arterial pressure (mmHg) were recorded at baseline (start of landiolol administration or respective index time point in the NL-group), and at 30, 60, 90, 120, and 180 min thereafter. In each line chart, the red line marks the median value over the displayed time interval. The gray dots and lines depict the individual patient values and their changes over the follow-up period. * indicates a statistically significant difference between groups (*p* < 0.05).

At the initiation of landiolol therapy in the treatment group, or at the respective index time point in the standard therapy group (representing the hypothetical timing of landiolol initiation), the median (IQR) HR was 135.0 (120.0–147.0) beats per minute. The median (IQR) systolic blood pressure was 94.0 (78.0–114.0) mmHg, and the median (IQR) diastolic blood pressure was 55.0 (47.0–63.0) mmHg. After 120 min, significantly lower systolic blood pressure (median 107.0 vs. 92.0 mmHg, mean 108.1 vs. 90.3 mmHg; *p* = 0.021) and MAP (median 74.0 vs. 70.0 mmHg, mean 78.9 vs. 67.0 mmHg; *p* = 0.027) were observed in the L-group; however, these differences were compensated by the end of follow-up. No statistically significant differences were observed in the remaining parameters during the first 3 h following initiation of treatment (Figure 1 and Table 4). In the L-group, one patient presented with severe hemodynamic instability, characterized by a HR of 171 bpm and a systolic blood pressure of 76 mmHg despite receiving high-dose catecholaminergic support with norepinephrine at 25 mL/h (equivalent to 5 mg/h) upon admission to the intensive care unit. The patient experienced fatal outcome 180 min after treatment initiation with landiolol.

### 3.5. Primary and Secondary Endpoints

The primary composite endpoint was achieved in four patients (40.0%) in the L-group and in 21 patients (51.2%) in the NL-group (*p* = 0.525). Patients treated with landiolol exhibited a median HR reduction of 25.3% from baseline within the first 3 h. In comparison, patients in the NL-group showed a smaller mean HR reduction of 21.9% over the same 180 min period (*p* < 0.001) (Table 4).

### 3.6. Regression Analysis

Multivariate logistic regression analysis did not identify any significant independent predictors for achieving the primary composite endpoint, defined as HR reduction with maintenance of mean systolic blood pressure above 65 mmHg. Notably, there was no statistically significant difference between patients who received landiolol and those who did not, as shown in Table 5.

## 4. Discussion

AF is a frequent complication in critically ill patients and is associated with significant clinical deterioration due to its hemodynamic impact and thromboembolic risk [10]. In the ICU setting, rhythm control is often not feasible due to the presence of multiple comorbidities and underlying structural or functional cardiac alterations [23]. Pharmacological treatment options for AF are limited by substantial proarrhythmic and extracardiac side effects, particularly in unstable patients [12]. In this context, the ultra-short-acting, highly β_1_-selective, beta-blocker landiolol, offers an alternative for rate control [21,24], and this study investigates its efficacy, safety and added benefit over standard care in a highly selected ICU patient cohort with AF.

In our analysis, patients with AF admitted to the ICU were divided into those treated with landiolol (L-group) and those managed with conventional therapy (NL-group). The cohort included both male and female patients, with females accounting for slightly less than half of the population. Age distribution was similar across both groups, consistent with previous studies reporting a median age of around 70 to 75 years at AF diagnosis in critically ill populations [25,26]. Approximately half of the cohort demonstrated reduced left ventricular function, and the leading reasons for ICU admission of these medical, non-surgical patients were septic and cardiogenic shock. These findings align with existing literature identifying these shock states and underlying cardiac dysfunction as key contributors to AF onset in the ICU and underscore the clinical complexity where standard rate control therapies frequently fail to achieve adequate stabilization [10,27]. Altogether, the profile of this cohort underscores the complexity and clinical instability commonly encountered in the ICU, highlighting the need for individualized rate control strategies and the potential necessity for alternative pharmacological interventions.

At the time of ICU admission, vital parameters, including MAP, HR, respiratory rate, SpO_2_, PaO_2_, and the Horovitz index, did not significantly differ between the analyzed patient groups, suggesting a comparable baseline severity of the clinical condition. The elevated heart and respiratory rates observed across the cohort are consistent with the acute hemodynamic stress and respiratory compromise often reported in critically ill patients [28].

A substantial proportion of patients required catecholamine support, highlighting the hemodynamic instability frequently encountered in this population [29,30]. Attempts at rhythm control through pharmacological therapy and electrical cardioversion were not successful, underscoring the challenges associated with refractory arrhythmias in critically ill patients, where underlying metabolic, ischemic, and inflammatory disturbances may significantly impair the recovery of SR [11,31]. Failure of standard rate control therapy was a frequent clinical scenario in this cohort, necessitating additional options for rapid stabilization. Overall, the management of these patients required a multifaceted approach involving vasoactive agents, antiarrhythmic therapy, and, when indicated, fluid resuscitation. These findings align with current guidelines advocating individualized hemodynamic support strategies based on continuous reassessment of fluid responsiveness and cardiac function [32].

At the time of therapy initiation, the patients exhibited elevated HRs and low systolic and diastolic blood pressures, reflecting significant hemodynamic compromise associated with the arrhythmia. Although a transient decrease in systolic and MAPs was observed in the L-group after two hours, no further significant hemodynamic differences between the groups were detected during the subsequent observation period. One patient in the L-group experienced severe hemodynamic deterioration leading to death; however, the patient had already been admitted to the ICU in a state of profound circulatory compromise despite high-dose catecholaminergic therapy. In our opinion, the adverse event is more likely attributable to the patient’s pre-existing hemodynamic compromise rather than to a direct causal effect of landiolol administration. Nevertheless, careful hemodynamic monitoring during both initiation and continuation of the landiolol therapy remains essential, as highlighted by Nishikawa et al., who reported transient blood pressure decreases in 4% of patients, isolated cases of cardiac arrest, and decreased ejection fraction, each occurring in approximately 1% of treated individuals [33]. Moreover, these findings reinforce the importance of individualized treatment decisions when introducing negative chronotropic agents in hemodynamically unstable patients [11].

Regarding clinical efficacy, the achievement of the predefined composite endpoint, HR control while maintaining sufficient systolic blood pressure, did not significantly differ between groups. Furthermore, multivariate logistic regression analysis did not identify independent predictors for successful endpoint achievement, emphasizing the multifactorial nature of therapeutic response in this population. Importantly, however, landiolol administration after failed standard rate control therapy resulted in a significantly greater reduction in HR compared to conventional therapy alone. This observation suggests that landiolol provides an effective additional option for improving HR control in cases where conventional strategies have been insufficient.

Previous studies have reported the efficacy and safety of landiolol in different clinical settings, particularly in postoperative or cardiac surgery patients [34,35]. However, evidence in critically ill medical ICU patients with refractory AF remains scarce. Our study therefore extends the current knowledge by highlighting the potential role of landiolol beyond perioperative use, broadening its application to a wider critically ill population. Thus, landiolol may close an important therapeutic gap in the management of critically ill patients with refractory AF, not only in new-onset cases, as reported by Levy et al., but also in those with preexisting AF [21]. Nevertheless, whether this pharmacodynamic effect confers a meaningful clinical benefit remains uncertain, given the multifactorial nature of hemodynamic stabilization in this population.

### Limitations

Several limitations of this study should be acknowledged. First, the cohort was small, reflecting the highly selected nature of this critically ill ICU population, consisting of patients with significant hemodynamic deterioration secondary to inadequate rate control in AF. As such, the limited number of eligible patients inherently restricted the sample size and may have limited the statistical power to detect differences in clinical outcomes beyond HR control. Given the pilot design and the imbalanced sample, the minimal detectable standardized effect at 80% power (two-sided α = 0.05) was d ≈ 0.99; thus, the study is powered only for large effects. To mitigate this limitation, regression analyses adjusting for relevant covariates were performed, and effect sizes with 95% confidence intervals are reported, while *p*-values are interpreted cautiously. In addition, the observational design without randomization introduces a potential for selection bias and unmeasured confounding variables that could have influenced the results. The short observation period of three hours primarily reflects immediate hemodynamic effects and does not allow conclusions regarding longer-term safety or efficacy.

## 5. Conclusions

In our study, landiolol administration after failed conventional therapy led to a significantly greater reduction in HR compared to conventional care in critically ill patients. This additional rate control was achieved even after standard therapy failure and without compromising hemodynamic stability. These results suggest that landiolol may offer an effective and hemodynamically safe add-on option for HR management in critically ill patients with refractory AF. However, larger randomized studies are needed to confirm these findings and to determine whether improved HR control translates into better clinical outcomes.

## Figures and Tables

**Table 1:** Baseline characteristics of the patients in both groups.

Baseline Characteristics	Total (*n* = 51)	L-Group (*n* = 10)	NL-Group (*n* = 41)	*p*-Value
Age, *median* (*IQR*)	69.0 (64.0–79.0)	66.0 (57.5–79.2)	70.0 (65.5–79.5)	0.392
Female, *n* (%)	22.0 (43.1)	5.0 (50.0)	17.0 (41.5)	0.728
Male, *n* (%)	29.0 (56.9)	5.0 (50.0)	24.0 (58.5)	
Invasive mechanical ventilation, *n* (%)	38.0 (74.5)	8.0 (80.0)	30.0 (73.2)	1.000
APACHE II score, *median* (*IQR*)	26.0 (18.5–30.0)	25.0 (22.0–28.0)	27.0 (17.7–30.0)	0.930
First diagnosis of AF, *n* (%)	28.0 (54.9)	5.0 (50.0)	23.0 (56.1)	0.728
Paroxysmal AF, *n* (%)	6.0 (11.8)	3.0 (30.0)	3.0 (7.3)	0.050
Persistent AF, *n* (%)	12.0 (23.5)	2.0 (20.0)	10.0 (24.4)	0.769
Permanent AF, *n* (%)	5.0 (9.8)	0	5.0 (12.2)	0.245
Normal LVEF, *n* (%)	23.0 (45.1)	3.0 (30.0)	20.0 (48.8)	0.480
Mildly reduced LVEF, *n* (%)	7.0 (13.7)	1.0 (10.0)	6.0 (14.6)	1.000
Moderately reduced LVEF, *n* (%)	5.0 (9.8)	2.0 (20.0)	3.0 (7.3)	0.250
Severely reduced LVEF, *n* (%)	16.0 (31.4)	4.0 (40.0)	12.0 (29.3)	0.705
Cardiopulmonary resuscitation, *n* (%)	10.0 (19.6)	1.0 (10.0)	9.0 (22.0)	0.664
Cardiogenic shock, *n* (%)	17.0 (33.3)	4.0 (40.0)	13.0 (31.7)	0.714
STEMI, *n* (%)	6.0 (11.8)	1.0 (10.0)	5.0 (12.2)	1.000
NSTEMI, *n* (%)	1.0 (2.0)	0	1.0 (2.4)	1.000
Primary rhythm disorder, *n* (%)	5.0 (9.8)	0	5.0 (12.2)	0.569
Myocarditis, *n* (%)	1.0 (2.0)	1.0 (10.0)	0	0.196
Pulmonary embolism, *n* (%)	1.0 (2.0)	1.0 (10.0)	0	0.196
Septic shock, *n* (%)	35.0 (68.6)	6.0 (60.0)	29.0 (70.7)	0.705
Gastrointestinal bleeding, *n* (%)	1.0 (2.0)	1.0 (10.0)	0	0.196
Sodium [mmol/L], *median (IQR)*	142.0 (138.0–144.0)	143.0 (139.5–144.5)	142.0 (137.5–143.5)	0.424
Potassium [mmol/L], *median (IQR)*	4.1 (3.8–4.7)	4.3 (4.1–4.9)	4.1 (3.8–4.6)	0.147
Creatinine [µmol/L], *median (IQR)*	142.0 (95.0–229.0)	139.5 (122.5–233.0)	142.0 (93.5–219.5)	0.785
Leukocytes [10^9^/L], *median (IQR)*	13.4 (7.7–21.1)	11.7 (7.8–24.2)	14.7 (6.8–20.6)	0.849

AF, atrial fibrillation; IQR, interquartile range (25th–75th percentile); L-group, landiolol group; LVEF, left ventricular ejection fraction; NL-group, no landiolol group; NSTEMI, non–ST-elevation myocardial infarction; STEMI, ST-elevation myocardial infarction.

**Table 2:** Vital parameters at ICU admission in both groups.

Vital Parameters at ICU Admission	Total (*n* = 51)	L-Group (*n* = 10)	NL-Group (*n* = 41)	*p*-Value
Heart rate [beats/min], *median (IQR)*	160.0 (142.0–175.0)	168.0 (152.7–174.5)	158.0 (141.5–175.5)	0.349
Respiratory rate [breaths/min], *median (IQR)*	24.0 (20.0–33.3)	22.5 (20.7–25.0)	24.5 (17.7–37.7)	0.524
Mean arterial pressure [mmHg], *median (IQR)*	67.0 (60.0–75.0)	70.0 (64.5–76.5)	66.0 (59.5–78.0)	0.462
Temperature [°C], *median (IQR)*	37.2 (36.2–37.8)	37.5 (36.9–38.5)	37.1 (36.1–37.7)	0.092
pH (arteriell), *median (IQR)*	7.3 (7.2–7.4)	7.3 (7.2–7.4)	7.3 (7.2–7.4)	0.868
PaO_2_ [mmHg], *median (IQR)*	86.0 (74.0–100.0)	93.5 (84.0–111.2)	83.0 (72.0–96.0)	0.085
SpO_2_ [%], *median (IQR)*	95.0 (92.5–97.0)	96.0 (93.2–97.2)	95.0 (92.0–96.0)	0.412
Horovitz index, *median (IQR)*	155.0 (113.7–215.0)	164.0 (126.5–248.7)	150.5 (106.0–213.2)	0.235

ICU, intensive care unit; IQR, interquartile range (25th–75th percentile); L-group, landiolol group; min, minutes; mmHg, millimeters of mercury; NL-group, no landiolol group; PaO_2_, arterial oxygen pressure; pH, potential of hydrogen; SpO_2_, oxygen saturation.

**Table 3:** Conventional treatment of the patients in both groups.

Conventional Treatment	Total (*n* = 51)	L-Group (*n* = 10)	NL-Group (*n* = 41)	*p*-Value
Electrical cardioversion, *n* (%)	20.0 (39.2)	6.0 (60.0)	14.0 (34.1)	0.163
Amiodaron bolus therapy, *n* (%)	23.0 (45.1)	6.0 (60.0)	17.0 (41.5)	0.316
Amiodaron continuous therapy, *n* (%)	1.0 (2.0)	0	1.0 (2.4)	1.000
Digoxin therapy, *n* (%)	13.0 (25.5)	2.0 (20.0)	11.0 (26.8)	1.000
Metoprolol bolus therapy, *n* (%)	3.0 (5.9)	0	3.0 (7.3)	1.000
Metoprolol bolus and continuous therapy, *n* (%)	2.0 (3.9)	0	2.0 (4.9)	1.000
Combined Digoxin and Amiodaron therapy, *n* (%)	6.0 (11.8)	0	6.0 (14.6)	0.331
Intensified infusion therapy, *n* (%)	22.0 (43.1)	1.0 (10.0)	21.0 (51.2)	0.030
Epinephrine therapy, *n* (%)	4.0 (7.8)	0	4.0 (9.8)	0.573
Norepinephrine therapy, *n* (%)	32.0 (62.7)	7.0 (70.0)	25.0 (61.0)	0.725

L-group, landiolol group; NL-group, no landiolol group.

**Table 4:** Hemodynamic changes within the first three hours of treatment.

Hemodynamic Parameters	Total (*n* = 51)	L-Group (*n* = 10)	NL-Group (*n* = 41)	*p*-Value
HR initial [bpm], *median (IQR)*	160.0 (144.0–176.0)	168.0 (152.7–174.5)	160.0 (142.5–176.5)	0.610
HR 30 min [bpm], *median (IQR)*	135.0 (120.0–147.0)	137.0 (122.2–141.2)	134.0 (119.0–151.5)	0.794
HR 60 min [bpm], *median (IQR)*	133.0 (117.0–145.0)	126.0 (113.0–136.7)	134.0 (118.0–148.0)	0.141
HR 90 min [bpm], *median (IQR)*	126.0 (110.0–142.0)	127.5 (119.0–136.7)	126.0 (103.5–146.5)	0.906
HR 120 min [bpm], *median (IQR)*	127.0 (110.0–146.0)	127.0 (112.2–130.0)	128.0 (107.5–149.5)	0.393
HR 180 min [bpm], *median (IQR)*	125.0 (98.0–142.0)	125.5 (81.0–140.0)	125.0 (98.5–142.0)	0.569
Sys ABP initial [mmHg], *median (IQR)*	94.0 (78.0–114.0)	93.0 (77.5–107.5)	94.0 (78.5–116.0)	0.669
Sys ABP 30 min [mmHg], *median (IQR)*	97.0 (87.0–114.0)	97.0 (70.7–116.0)	95.0 (87.0–114.5)	0.849
Sys ABP 60 min [mmHg], *median (IQR)*	97.0 (84.0–116.0)	97.5 (68.7–112.7)	97.0 (85.0–117.0)	0.491
Sys ABP 90 min [mmHg], *median (IQR)*	109.0 (94.0–119.0)	99.0 (86.2–116.2)	109.0 (96.0–120.0)	0.217
Sys ABP 120 min [mmHg], *median (IQR)*	104.0 (93.0–119.0)	92.0 (84.7–105.7)	107.0 (96.0–125.0)	0.021
Sys ABP 180 min [mmHg], *median (IQR)*	97.0 (88.0–113.0)	93.5 (84.0–105.0)	100.0 (88.5–115,5)	0.217
Dia ABP initial [mmHg], *median (IQR)*	55.0 (47.0–63.0)	54.5 (48.0–63.0)	55.0 (46.5–64.5)	0.868
Dia ABP 30 min [mmHg], *median (IQR)*	60.0 (53.0–64.0)	59.0 (51.5–70.0)	60.0 (53.0–64.0)	0.669
Dia ABP 60 min [mmHg], *median (IQR)*	59.0 (51.0–64.0)	60.5 (53.2–71.7)	59.0 (50.5–63.0)	0.318
Dia ABP 90 min [mmHg], *median (IQR)*	61.0 (54.0–66.0)	61.0 (57.0–66.7)	61.0 (54.0–66.5)	0.660
Dia ABP 120 min [mmHg], *median (IQR)*	59.0 (55.0–69.0)	56.5 (54.5–61.5)	59.0 (54.5–70.0)	0.235
Dia ABP 180 min [mmHg], *median (IQR)*	57.0 (52.0–63.0)	60.5 (44.0–63.5)	57.0 (52.5–62.5)	0.972
Mean ABP initial [mmHg], *median (IQR)*	68.0 (60.0–80.0)	67.0 (58.5–76.5)	68.0 (59.5–80.5)	0.749
Mean ABP 30 min [mmHg], *median (IQR)*	71.0 (63.0–80.0)	72.0 (60.2–81.5)	70.0 (63.0–78.5)	0.953
Mean ABP 60 min [mmHg], *median (IQR)*	70.0 (64.0–82.0)	73.0 (59.5–84.7)	69.0 (64.0–82.0)	0.896
Mean ABP 90 min [mmHg], *median (IQR)*	75.0 (68.0–83.0)	72.0 (68.2–80.5)	76.0 (67.5–83.5)	0.521
Mean ABP 120 min [mmHg], *median (IQR)*	74.0 (69.0–85.0)	70.0 (64.7–74.2)	74.0 (69.0–88.5)	0.027
Mean ABP 180 min [mmHg], *median (IQR)*	71.0 (64.0–78.0)	72.5 (54.5–75.7)	71.0 (65.0–79.5)	0.569

ABP, arterial blood pressure; bpm, beats per minute; Dia, diastolic; HR, heart rate; IQR, interquartile range (25th–75th percentile); min, minutes; Sys, systolic.

**Table 5:** Multivariate logistic regression analysis of potential predictors for achieving the primary composite endpoint.

	Regression Coefficient B	Standard Error	*p*-Value	OR (95% CI)
Age [years]	0	0	0.650	0.99 (0.94–1.04)
Female gender	0.5	0.6	0.400	1.66 (0.51–5.38)
Landiolol treatment	−0.7	0.8	0.330	0.47 (0.10–2.14)
Reduced LVEF	0.8	0.6	0.180	2.22 (0.69–7.17)

LVEF, left ventricular ejection fraction.

## Data Availability

The data presented in this study are available on request from the authors. The data are not publicly available due to data privacy laws.

## Abbreviations

The following abbreviations are used in this manuscript:

AF	Atrial fibrillation
Bpm	Beats per minute
CIP	Critically ill patients
e.g.	For example
HR	Heart rate
ICU	Intensive care unit
IQR	Interquartile range
L-group	Landiolol group
LVEF	Left ventricular ejection fraction
Min.	Minutes
MmHg	Millimeters of mercury
NL-group	No landiolol group
NSTEMI	Non–ST-elevation myocardial infarction
OAC	Oral anticoagulation
PaO_2_	Arterial oxygen pressure
pH	Potential of hydrogen
SpO_2_	Oxygen saturation
SR	Sinus rhythm
STEMI	ST-elevation myocardial infarction

## References

[1] Chauhan V.S., Krahn A.D., Klein G.J., Skanes A.C., Yee R. (2001). Supraventricular Tachycardia. Med. Clin. N. Am..

[2] Duby J.J., Heintz S.J., Bajorek S.A., Heintz B.H., Durbin-Johnson B.P., Cocanour C.S. (2017). Prevalence and Course of Atrial Fibrillation in Critically Ill Trauma Patients. J. Intensive Care Med..

[3] Seguin P., Signouret T., Laviolle B., Branger B., Mallédant Y. (2004). Incidence and Risk Factors of Atrial Fibrillation in a Surgical Intensive Care Unit. Crit. Care Med..

[4] Maisel W.H., Rawn J.D., Stevenson W.G. (2001). Atrial Fibrillation after Cardiac Surgery. Ann. Intern. Med..

[5] Zakynthinos G.E., Tsolaki V., Xanthopoulos A., Karavidas N., Vazgiourakis V., Bardaka F., Giamouzis G., Pantazopoulos I., Makris D. (2024). Prevalence, Risk Factors, and Mortality of New-Onset Atrial Fibrillation in Mechanically Ventilated Critically Ill Patients. J. Clin. Med..

[6] Bosch N.A., Cimini J., Walkey A.J. (2018). Atrial Fibrillation in the ICU. Chest.

[7] Walkey A.J., Hogarth D.K., Lip G.Y.H. (2015). Optimizing Atrial Fibrillation Management: From ICU and Beyond. Chest.

[8] Reyes L.F., Restrepo M.I., Hinojosa C.A., Soni N.J., Anzueto A., Babu B.L., Gonzalez-Juarbe N., Rodriguez A.H., Jimenez A., Chalmers J.D. (2017). Severe Pneumococcal Pneumonia Causes Acute Cardiac Toxicity and Subsequent Cardiac Remodeling. Am. J. Respir. Crit. Care Med..

[9] Brown A.O., Mann B., Gao G., Hankins J.S., Humann J., Giardina J., Faverio P., Restrepo M.I., Halade G.V., Mortensen E.M. (2014). Streptococcus Pneumoniae Translocates into the Myocardium and Forms Unique Microlesions That Disrupt Cardiac Function. PLoS Pathog..

[10] Klouwenberg P.M.C.K., Frencken J.F., Kuipers S., Ong D.S.Y., Peelen L.M., van Vught L.A., Schultz M.J., van der Poll T., Bonten M.J., Cremer O.L. (2017). Incidence, Predictors, and Outcomes of New-Onset Atrial Fibrillation in Critically Ill Patients with Sepsis. A Cohort Study. Am. J. Respir. Crit. Care Med..

[11] Van Gelder I.C., Rienstra M., Bunting K.V., Casado-Arroyo R., Caso V., Crijns H.J.G.M., De Potter T.J.R., Dwight J., Guasti L., Hanke T. (2024). 2024 ESC Guidelines for the Management of Atrial Fibrillation Developed in Collaboration with the European Association for Cardio-Thoracic Surgery (EACTS): Developed by the Task Force for the Management of Atrial Fibrillation of the European Society of Cardiology (ESC), with the Special Contribution of the European Heart Rhythm Association (EH-RA) of the ESC. Endorsed by the European Stroke Organisation (ESO). Eur. Heart J..

[12] January C.T., Wann L.S., Calkins H., Chen L.Y., Cigarroa J.E., Cleveland J.C., Ellinor P.T., Ezekowitz M.D., Field M.E., Furie K.L. (2019). 2019 AHA/ACC/HRS Focused Update of the 2014 AHA/ACC/HRS Guideline for the Management of Patients With Atrial Fibrillation: A Report of the American College of Cardiology/American Heart Association Task Force on Clinical Practice Guidelines and the Heart Rhythm Society in Collaboration With the Society of Thoracic Surgeons. Circulation.

[13] Markman T.M., Jarrah A.A., Tian Y., Mustin E., Guandalini G.S., Lin D., Epstein A.E., Hyman M.C., Deo R., Supple G.E. (2022). Safety of Pill-in-the-Pocket Class 1C Antiarrhythmic Drugs for Atrial Fibrillation. JACC Clin. Electrophysiol..

[14] Hussein M.T.E., Pamnani P. (2024). Pill-in-the-Pocket for Paroxysmal Atrial Fibrillation: A Review and Case Study. J. Nurse Pract..

[15] Campbell T.J., Williams K.M. (1998). Therapeutic Drug Monitoring: Antiarrhythmic Drugs. Br. J. Clin. Pharmacol..

[16] Carlisle M.A., Fudim M., DeVore A.D., Piccini J.P. (2019). Heart Failure and Atrial Fibrillation, Like Fire and Fury. JACC Heart Fail..

[17] Koniari I., Artopoulou E., Velissaris D., Mplani V., Anastasopoulou M., Kounis N., De Gregorio C., Tsigkas G., Karu-nakaran A., Plotas P. (2022). Pharmacologic Rate versus Rhythm Control for Atrial Fibrillation in Heart Failure Patients. Medicina.

[18] Yoshida Y., Terajima K., Sato C., Akada S., Miyagi Y., Hongo T., Takeda S., Tanaka K., Sakamoto A. (2008). Clinical Role and Efficacy of Landiolol in the Intensive Care Unit. J. Anesth..

[19] Iguchi S., Iwamura H., Nishizaki M., Hayashi A., Senokuchi K., Kobayashi K., Sakaki K., Hachiya K., Ichioka Y., Kawamura M. (1992). Development of a Highly Cardioselective Ultra Short-Acting β-Blocker, ONO-1101. Chem. Pharm. Bull..

[20] Murakami M., Furuie H., Matsuguma K., Wanibuchi A., Kikawa S., Irie S. (2005). Pharmacokinetics and Pharmacodynamics of Landiolol Hydrochloride, an Ultra Short-Acting β1-Selective Blocker, in a Dose Escalation Regimen in Healthy Male Volunteers. Drug Metab. Pharmacokinet..

[21] Levy B., Slama M., Lakbar I., Maizel J., Kato H., Leone M., Okada M. (2024). Landiolol for Treatment of New-Onset Atrial Fibrillation in Critical Care: A Systematic Review. J. Clin. Med..

[22] Si X., Yuan H., Shi R., Song W., Guo J., Jiang J., Yang T., Ma X., Wang H., Chen M. (2025). Comparison of the Efficacy and Safety of Landiolol and Esmolol in Critically Ill Patients: A Propensity Score-Matched Study. Ann. Intensive Care.

[23] Arrigo M., Bettex D., Rudiger A. (2014). Management of Atrial Fibrillation in Critically Ill Patients. Crit. Care Res. Pract..

[24] Domanovits H., Wolzt M., Stix G. (2018). Landiolol: Pharmacology and Its Use for Rate Control in Atrial Fibrillation in an Emergency Setting. Eur. Heart J. Suppl..

[25] Wells G.L., Morris P.E. (2011). Incidence and Prognosis of Atrial Fibrillation in Patients with Sepsis. Cardiol. Res..

[26] Rottmann F.A., Abraham H., Welte T., Westermann L., Bemtgen X., Gauchel N., Supady A., Wengenmayer T., Staudacher D.L. (2024). Atrial Fibrillation and Survival on a Medical Intensive Care Unit. Int. J. Cardiol..

[27] Kuipers S., Klouwenberg P.M.K., Cremer O.L. (2014). Incidence, Risk Factors and Outcomes of New-Onset Atrial Fibrillation in Patients with Sepsis: A Systematic Review. Crit. Care.

[28] Singer M., Deutschman C.S., Seymour C.W., Shankar-Hari M., Annane D., Bauer M., Bellomo R., Bernard G.R., Chiche J.-D., Coopersmith C.M. (2016). The Third International Consensus Definitions for Sepsis and Septic Shock (Sepsis-3). JAMA.

[29] Annane D., Vignon P., Renault A., Bollaert P.-E., Charpentier C., Martin C., Troché G., Ricard J.-D., Nitenberg G., Papazian L. (2007). Norepinephrine plus Dobutamine versus Epinephrine Alone for Management of Septic Shock: A Randomised Trial. Lancet.

[30] Vincent J.-L., Backer D.D. (2013). Circulatory Shock. N. Engl. J. Med..

[31] Fuster V., Rydén L.E., Asinger R.W., Cannom D.S., Crijns H.J., Frye R.L., Halperin J.L., Kay G.N., Klein W.W., Lévy S. (2001). ACC/AHA/ESC Guidelines for the Management of Patients with Atrial Fibrillation: Executive Summary: A Report of the American College of Cardiology/American Heart Association Task Force on Practice Guidelines and the European Society of Cardiology Committee for Practice Guidelines and Policy Conferences (Committee to Develop Guide-lines for the Management of Patients With Atrial Fibrillation). J. Am. Coll. Cardiol..

[32] Evans L., Rhodes A., Alhazzani W., Antonelli M., Coopersmith C.M., French C., Machado F.R., Mcintyre L., Ostermann M., Prescott H.C. (2021). Surviving Sepsis Campaign: International Guidelines for Management of Sepsis and Septic Shock 2021. Crit. Care Med..

[33] Kakihana Y., Nishida O., Taniguchi T., Okajima M., Morimatsu H., Ogura H., Yamada Y., Nagano T., Morishima E., Matsuda N. (2020). Efficacy and Safety of Landiolol, an Ultra-Short-Acting Β1-Selective Antagonist, for Treatment of Sepsis-Related Tachyarrhythmia (J-Land 3S): A Multicentre, Open-Label, Randomised Controlled Trial. Lancet Respir. Med..

[34] Sakamoto A., Kitakaze M., Takamoto S., Namiki A., Kasanuki H., Hosoda S., JL-KNIGHT Study Group (2012). Landiolol, an Ultra-Short-Acting Β_1_-Blocker, More Effectively Terminates Atrial Fibrillation than Diltiazem after Open Heart Surgery: Prospective, Multicenter, Randomized, Open-Label Study (JL-KNIGHT Study). Circ. J..

[35] Cafaro T., Allwood M., McIntyre W.F., Park L.J., Daza J., Ofori S.N., Ke Wang M., Borges F.K., Conen D., Marcucci M. (2023). Landiolol for the Prevention of Postoperative Atrial Fibrillation after Cardiac Surgery: A Systematic Review and Meta-Analysis. Can. J. Anaesth..

